# Intracellular Localization during Blood–Brain Barrier Crossing Influences Extracellular Release and Uptake of Fluorescent Nanoprobes

**DOI:** 10.3390/nano13131999

**Published:** 2023-07-03

**Authors:** Ornella Muscetti, Naym Blal, Valentina Mollo, Paolo Antonio Netti, Daniela Guarnieri

**Affiliations:** 1 Center for Advanced Biomaterials for Healthcare, Istituto Italiano di Tecnologia (IIT@CRIB), Largo Barsanti e Matteucci 53, 80125 Naples, Italy; ornella.muscetti@gmail.com (O.M.); nblal@unisa.it (N.B.); valentina.mollo@iit.it (V.M.); nettipa@unina.it (P.A.N.); 2 Dipartimento di Chimica e Biologia “Adolfo Zambelli”, Università degli Studi di Salerno, Via Giovanni Paolo II 132, 84084 Salerno, Italy; 3 Interdisciplinary Research Centre on Biomaterials, (CRIB), University of Naples Federico II, 80125 Naples, Italy; 4 Department of Chemical Materials and Industrial Production (DICMaPI), University of Naples Federico II, 80125 Naples, Italy

**Keywords:** blood–brain barrier, endocytosis, excretion, brain tumor, fluorescent nanoparticles

## Abstract

To improve the efficacy of nanoparticles (NPs) and boost their theragnostic potential for brain diseases, it is key to understand the mechanisms controlling blood–brain barrier (BBB) crossing. Here, the capability of 100 nm carboxylated polystyrene NPs, used as a nanoprobe model, to cross the human brain endothelial hCMEC/D3 cell layer, as well as to be consequently internalized by human brain tumor U87 cells, is investigated as a function of NPs’ different intracellular localization. We compared NPs confined in the endo-lysosomal compartment, delivered to the cells through endocytosis, with free NPs in the cytoplasm, delivered by the gene gun method. The results indicate that the intracellular behavior of NPs changed as a function of their entrance mechanism. Moreover, by bypassing endo-lysosomal accumulation, free NPs were released from cells more efficiently than endocytosed NPs. Most importantly, once excreted by the endothelial cells, free NPs were released in the cell culture medium as aggregates smaller than endocytosed NPs and, consequently, they entered the human glioblastoma U87 cells more efficiently. These findings prove that intracellular localization influences NPs’ long-term fate, improving their cellular release and consequent cellular uptake once in the brain parenchyma. This study represents a step forward in designing nanomaterials that are able to reach the brain effectively.

## 1. Introduction

In the last several decades, the use of nanoparticles (NPs) has widely grown in the biological and biomedical fields, especially for diagnostic and drug delivery purposes. In fact, thanks to their size, shape, and surface properties, NPs can be engineered ad hoc for targeting specific tissues, and they represent an ideal candidate to overcome biological barriers [1,2,3]. Among others, NPs have been proven to be able to cross the blood–brain barrier (BBB) and accumulate in the central nervous system (CNS) [4,5,6]. In fact, due to the presence of tight junctions, the BBB remains the main obstacle to deliver contrast agents and drugs for the diagnosis and treatment of CNS-related pathologies [7,8]. Therefore, NPs represent a valid alternative to the invasive techniques currently used to administrate therapies to the brain [9,10,11].

Many efforts have been made for designing and engineering nanomaterials for CNS theragnostic applications, and extensive research has been focused on elucidating the mechanisms used by cells to internalize NPs both in vivo and in vitro [12,13,14]. Nevertheless, the use of NPs in nanomedicine presents some limits. In fact, it is widely reported that most NPs enter the cell by endocytosis [15,16]. The endocytic mechanism leads to NP accumulation along the endo-lysosomal pathway [17,18]. As a consequence, there is a decrease in the ability of NPs to cross the BBB [19] and reach the brain parenchyma [20,21]. Furthermore, their accumulation in the lysosomal compartment causes, in some cases, cytotoxic effects due to the low pH and degradative environment [22,23].

On the other hand, few data are available about the long-term fate of NPs excreted from the BBB. It has been reported that, for the endocytosis process, NP excretion from cells can be affected by several parameters [24]. For instance, it has been shown that the exocytosis process is size-dependent. In fact, Serda and colleagues showed that the exocytosis of super-paramagnetic iron oxide NPs (SPIONs) trapped in porous silicon carriers from the murine macrophage J774 cell line was significantly greater for carriers containing 15 nm SPIONs than carriers loaded with 30 nm NPs at the same concentration [25]. Moreover, 100 nm polystyrene NPs were more difficult to excrete than 50 nm polystyrene NPs from human lung cells [26]. In addition to their size, the NP shape is also important for exocytosis processes. As an example, the fraction of rod-shaped transferrin-coated gold NPs released from the HeLa and SNB 19 cell lines was much higher than sphere-shaped NPs [27]. Additionally, different surface modifications have important consequences on the exocytosis profile of NPs. In fact, gold NPs, functionalized with a non-targeting or targeting peptide, but with the same physicochemical properties (i.e., a size of ~35 nm and a zeta potential of ~−18 mV), showed different exocytosis profiles. The non-targeted NPs were re-uptaken by the cells after 4 h, while for a period of 6 h, the targeted NPs were progressively exocytosed [28]. Moreover, it was demonstrated that surface decoration with membranotropic virus-derived peptide gH625 enhanced the BBB crossing of aminated polystyrene NPs in vitro [29]. Finally, the exocytosis kinetics of NPs is influenced by cell types as well. Recently, Liu and co-workers demonstrated that the exocytosis kinetic parameters strongly depended on the cell types but were insensitive to the initial intracellular concentration of carbon dots [30].

However, despite many efforts, the findings regarding NPs’ release mechanisms and fate after their experience in the intracellular BBB environment remain unclear and elusive [24]. In fact, the physicochemical properties of NPs’ surfaces as well as their colloidal stability can change during their uptake, lysosomal, and exocytosis processing [24,31,32]. Therefore, excreted NPs may exhibit a different surface and aggregation state after their cellular release. Understanding the mechanisms of NP cellular release is particularly relevant, not only to predict the efficiency of NPs to cross the BBB, but also to assess the capability of excreted NPs to be internalized by target cells that are in the brain parenchyma. Such investigations may provide important insights into the design of efficient and safe nanoprobes and drug-loaded nanocarriers, thus boosting their theragnostic potential. 

Our hypothesis is that the main limit of NP excretion is represented by the propensity of NPs to accumulate in the endo-lysosome compartment. From this perspective, we previously demonstrated that by bypassing the endo-lysosomal pathway using a pneumatic method, namely the gene gun, it was possible to obtain free NPs in the cytoplasm in several cell lines [23]. Hence, shot NPs prevalently showed a random walk behavior, typical of non-vesicle confined NPs, and no co-localization with lysosomes. Furthermore, cytoplasmic localization reduced the cytotoxicity of some NPs significantly [23,33].

In this work, we analyzed and compared the excretion profile of endocytosed (endo) and shot NPs from human brain endothelial cells cultured in standard and Transwell cell culture systems, as in vitro models of the human BBB, to shed light on the extracellular fate of NPs after their passage through the BBB. In particular, we delivered 100 nm carboxylated polystyrene NPs, chosen as a commercially available nanoprobe model, to the endo-lysosomal compartment by the endocytosis mechanism and directly to the cytosol by the gene gun method in the in vitro model of the BBB, composed of human brain endothelial hCMEC/D3 cells. We analyzed in detail the effects of the different intracellular environments (endo-lysosomes and cytosol) on the excretion profile of the NPs. In addition, we compared the obtained results with those obtained with nanoparticles functionalized with the cell-penetrating peptide gH625 able to escape the endo-lysosomal compartment [29,33]. Moreover, we preliminarily characterized the aggregation state and the amount of protein corona of the released NPs as a function of the NP intracellular localization. Furthermore, these parameters were correlated with the capability of excreted NPs to be taken up by the human glioblastoma U87 cell line as a model of brain tumor tissue and, hence, as a possible target for nanomaterials in the CNS. 

## 2. Materials and Methods

### 2.1. Nanoparticles

FluoSpheres yellow-green carboxylate-modified microspheres (NPs) with a diameter of 100 nm were purchased by Invitrogen (Thermo Fisher, Waltham, MA, USA). 

### 2.2. Cell Cultures

The immortalized human cerebral microvascular endothelial cells/clone D3 (hCMEC/D3) were kindly provided by Dr. P.-O. Couraud (INSERMU1016 UMR 8104, Institut Cochin, Paris, France). The hCMEC/D3 cells were cultured in EBM-2 medium (Lonza) supplemented with ascorbic acid (Sigma Aldrich, St. Louis, MO, USA), hydrocortisone (Sigma Aldrich), basic fibroblast growth factor (Sigma Aldrich), fetal bovine serum (FBS) (Gibco, Grand Island, NY, USA), and HEPES (Invitrogen, Waltham, MA, USA). Cells were grown on 100 mm diameter Petri dishes coated with rat tail collagen type-I (BD Biosciences, San Jose, CA, USA) at 37 °C with 5% CO_2_, 95% air, and saturated humidity. Cells used for the experiments were maintained between passage 23 and 33.

The immortalized human glioma cell line U87-MG (U87) were purchased from the American Type Culture Collection (ATCC, Manassas, VA, USA). Cells were maintained in DMEM supplemented with 10% heat-inactivated FBS and 100 units/mL penicillin/streptomycin at 37 °C in a humidified atmosphere of 5% CO_2_.

### 2.3. NP Intracellular Shooting by Gene Gun Method

A ballistic system (Gene Gun, BioRad, Hercules, CA, USA) was used to achieve free NPs in the cytoplasm as previously reported [33]. In brief, NPs were suspended in 30 µL of sterile MilliQ water at the final concentration of 1.8 × 10^12^ NPs/mL, deposited and left to dry on a rupture disk under a sterile hood. After solvent drying, 650 psi pressure was used to shoot NPs within cells, formerly seeded in 35 mm culture dishes at a density of 2 × 10^4^ cells per cm^2^. After shooting, cells were rinsed with PBS five times to remove non-internalized NPs. The cells were then harvested, counted, and reseeded on the opportune substrate for the specific experiments described below.

### 2.4. Quantification of Internalized Nanoparticles in hCMEC/D3 Cells

#### 2.4.1. Quantification of Endocytosed Nanoparticles

In order to evaluate the number of endocytosed NPs within cells, cells were roughly rinsed with PBS, after incubation of the NPs for 24 h, trypsinized, and counted in a Neubauer chamber. Cells were then centrifuged, and the pellets were lysed with lysis buffer (7 M urea, 2 M thiourea, 4% CHAPS, 30 mM Tris). To measure the amount of internalized NPs, cell lysates were analyzed with a spectrofluorometer (EnSpire, Perkin-Elmer, Waltham, MA, USA). Data were reported as the mean NP number normalized to the cell number.

#### 2.4.2. Quantification of Shot Nanoparticles

To evaluate the number of shot NPs within cells, the cells were shot with NPs by the gene gun, washed roughly with PBS, and then allowed to recover for 24 h at 37 °C. After recovery, cells were roughly rinsed with PBS, trypsinized, and counted in a Neubauer chamber. Afterward, cells were centrifuged, and the pellets were lysed with lysis buffer (7 M urea, 2 M thiourea, 4% CHAPS, 30 mM Tris). The amount of internalized NPs was measured by analyzing cell lysates with a spectrofluorometer (EnSpire, Perkin-Elmer). Data were reported as the mean NP number normalized to the number of cells.

### 2.5. Cytotoxicity Assay

Alamar blue assay was performed to evaluate cell viability of cells treated with shot and endocytosed NPs. The results of the assay were compared to non-treated cells, which were used as a control. For the assay, 3 × $10^{4}$ cells, seeded on a 35 mm cell culture dish, were incubated for 24 h with NP suspensions to allow endocytosis. On the other hand, for NP shooting, cells were shot with NPs by the gene gun, washed roughly with PBS, and then, allowed to recover for 24 h at 37 °C. After recovery, cells were trypsinized, counted, and seeded on fresh 35 mm cell culture dishes. Alamar blue assay was carried out according to the manufacturer’s procedure at 24, 48, and 72 h after NP exposure. Absorbance of Alamar blue reagent solution was read at 570 nm and 600 nm by a plate reader (Perkin-Elmer). Data were reported as the percentage of viable cells normalized to non-treated cells.

### 2.6. Co-Localization with Lysosomes

For co-localization tests, cells were rinsed twice with PBS after NP incubation, to remove non-internalized nanoparticles and fixed with 4% paraformaldehyde at room temperature (RT). After permeabilization and blocking, the lysosomes were localized with rabbit anti-LAMP 2 polyclonal primary antibodies (Abcam, Cambridge, UK) and with 568 goat anti-rabbit secondary antibodies (Molecular Probes, Invitrogen). All samples were then observed using a confocal microscope (SP5 Leica, Leica, Wetzlar, Germany) with a 63× oil immersion objective.

### 2.7. Excretion of Shot and Endocytosed Nanoparticles

To follow NP exocytosis process, 1 × $10^{4}$ cells were seeded into a 24-well plate and allowed to reach the confluence. Hence, cells were incubated with 100 nm NP suspension at the final concentrations of 3.60 × $10^{10}$ NP/mL in cell culture medium for 24 h at 37 °C. After NP incubation, cells were rinsed twice with PBS to remove non-internalized NPs and a fresh culture medium was added. The exocytosis of NPs was analyzed at different timepoints: 2, 24, 48, and 72 h. The amount of excreted NPs was monitored by measuring the content of NPs in both the cells and the medium as follows: at each time, cells were lysed with lysis buffer (7 M urea, 2 M thiourea, 4% CHAPS, 30 mM Tris) and cell lysate was analyzed by measuring fluorescence intensity (FI) at 488 nm excitation wavelength by spectrofluorometer (EnSpire, Perkin-Elmer). In order to measure the amount of exocytosed NPs, 100 µL of cell culture medium were collected and measured with spectrofluorometer. In order to quantify the amount of excreted NPs allowed to enter the cells by using gene gun method, 1 × $10^{4}$ cells were seeded on a 35 mm cell culture dish. After NP shooting, cells were trypsinized, counted, and reseeded in a 24-well plate. Excreted NPs were measured at the following timepoints: 0, 2, 24, 48, and 72 h. The amount of excreted NPs was measured both in cell and in medium by using a spectrofluorometer as described above. The number of released NPs was expressed as the percentage of the total number of NPs found in cell lysate and cell culture medium according to the following equation:

$$Released\;NPs\;\left(\%\right)=\left(\frac{Mean\;FI\;medium}{Mean\;FI\;cells+Mean\;FI\;medium}\right)\times 100$$

### 2.8. Excretion of Shot and Endocytosed Nanoparticles in Transwell Systems

Transcytosis experiments were carried out in order to evaluate the NP excretion profile on a Transwell system. Transwell permeable inserts (6.5 mm in diameter, 3 μm pore size; Corning) were pre-treated with rat tail type I collagen for 1 h at 37 °C in a dry incubator in order to allow the cell adhesion. After 24 h from NP endocytosis and shooting, 7 × 10^4^ hCMEC/D3 cells were seeded on a Transwell filter and incubated for 72 h at 37 °C. To assess the growth of cells on the porous inserts, transendothelial electrical resistance (TEER) was measured by using Millicell1-ERS Voltohmmeter (Millipore, Billerica, MA, USA). After 72 h incubation time, basal medium was collected and the amount of NPs was quantified by using a spectrofluorometer (EnSpire, Perkin-Elmer) at 488 nm wavelength. 

### 2.9. Transmission Electron Microscopy (TEM)

In order to precisely localize the intracellular nanoparticles in hCMEC/D3 cell line on Transwell, TEM analysis was performed. The samples were prepared as described in the following: after NP exposure, cells were trypsinized, reseeded on Transwell filter and fixed after 72 h. Afterward, Transwell containing confluent hCMEC/D3 cell monolayer was rinsed with PBS and fixed with a solution of 2.5% glutaraldehyde in 0.1 M sodium cacodylate buffer for 2 h at RT. Then, cells were post-fixed with 1% osmium tetroxide in 0.1 M sodium cacodylate buffer for 1 h at 4 °C, and dehydrated in graded concentrations of ethanol (30%, 50%, 70%, 95%, and 100%). Cells were then embedded in epoxy resin. Ultrathin sections were cut with an ultramicrotome (UC7, Leica). Sections of 90 nm were collected on copper grids and stained with lead citrate and uranyl acetate. The grids were visualized using a TEM Tecnai G2 (FEI Company, Hillsboro, OR, USA).

### 2.10. gH625 Peptide Synthesis, Nanoparticle Conjugation, and Characterization of gH625-NPs

gH625 peptide (Ac-HGLASTLTRWAHYNALIRAFGGG-COOH) were synthesized using the standard solid-phase-9-fluorenylmethoxycarbonyl (Fmoc) method as previously reported [29]. Good yields of 30–40% in purified peptide were obtained. 

Peptide conjugation to orange-fluorescent amine-modified polystyrene, 100 nm, nanoparticles (NPs) (Sigma-Aldrich) was performed as described previously [29]. Briefly, a solution of the peptide, EDC (1-Ethyl-3(3-dimethylamino-propyl)-carbodiimide, hydrochloride) and NHS (*N*-Hydroxysuccinimide) in a molar ratio of 4:4:1 was prepared in PBS buffer at pH 7.4, at RT under stirring for 30 min. NPs were conjugated with the preactivated-peptide in MES 0.1 M buffer at pH 5.5 for 3 h at RT in the presence of Tween 20. The yield of the reaction was higher than 90%. The peptide-NPs were purified from the unconjugated NPs by exclusion chromatography on a 1 × 18 cm Sephadex G-50 (Amersham Biosciences, Amersham, UK) column pre-equilibrated in PBS buffer at pH 7.4. The fluorescence spectra of peptide-NPs and unconjugated NPs were measured in a Cary Eclipse Varian fluorescence spectrophotometer (Varian Medical Systems, Palo Alto, CA, USA) in the same conditions. For 100 nm NPs, 35% of functionalization degree was used.

The gH625-NPs were characterized by circular dichroism (CD) and measurements of size and zeta-potential. CD spectra were recorded using a Jasco J-715 spectropolarimeter (Jasco, Hachioji-shi, Tokyo, Japan) in a 1.0 or 0.1 cm quartz cell at RT. The spectra were an average of 3 consecutive scans from 260 to 195 nm, recorded with a band width of 3 nm, a time constant of 16 s, and a scan rate of 10 nm/min. Spectra were recorded and corrected for the blank. Measurements of zeta potential and size of gH625-NPs were made with a Zetasizer Nano-ZS (Malvern Instruments, Worcestershire, UK). The measurements were conducted at 25 °C using a 3.6 × 10^10^ NP/mL suspension in MilliQ water at pH 7 (ionic strength 6 × 10^−5^ M). All measurements were performed in triplicate for each sample.

### 2.11. BCA Assay

The amount of adsorbed proteins on NP surface was determined by BCA assay (Sigma Aldrich). Briefly, 5 × 10^4^ cells were seeded on a 35 mm cell culture dish and incubated for 24 h with NP suspensions to allow endocytosis. Conversely, for NP shooting, cells were shot with NPs by gene gun method. After 24 h, endo and shot cells were washed roughly with PBS, trypsinized, counted, and seeded at a density of 7 × 10^4^ cells/cm^2^ on a Transwell filter with a pore size of 4 µm and incubated for 72 h at 37 °C. The Transwell filters were pre-treated with rat tail type I collagen for 1 h at 37 °C in a dry incubator in order to allow the cellular adhesion. Afterward, the basal medium was recovered, centrifuged, and the pellet was rinsed twice with PBS in order to remove cellular debris. Next, the pellet was suspended in 300 µL of sterilized water and was analyzed using a spectrofluorometer (EnSpire, Perkin-Elmer) in order to quantify the amount of recovered NPs. BCA assay was performed using the same number of NPs for endo and shot NPs according to the manufacturer’s procedure (Sigma Aldrich). Absorbance of BCA assay reagent solution was read at 560 nm by a plate reader spectrophotometer (EnSpire, Perkin-Elmer). Data were reported as µg/mL of protein normalized with non-conditioned NPs.

### 2.12. Quantification of Nanoparticles in U87 Cell Line

In order to evaluate the number of endocytosed NPs in brain tumor cells, 2 × 10^3^ U87 cells were seeded on a 96-well plate and incubated with NPs recovered from the basal media of hCMEC/D3 cell layers on Transwell systems after endocytosis and shooting at 4 × 10^6^ NPs/mL concentration. After incubation of the NPs for 72 h, cells were roughly rinsed with PBS and lysed with lysis buffer (7 M urea, 2 M thiourea, 4% CHAPS, 30 mM Tris). Cell lysates were analyzed using a spectrofluorometer (EnSpire, Perkin-Elmer) to measure the amount of internalized NPs. Data were reported as the number of NPs per cell. For confocal microscopy images, cells were seeded on 12 mm glass coverslips and incubated with 4 × 10^6^ NPs/mL for 72 h. Then, cells were washed with PBS to remove non-internalized NPs, fixed with 4% paraformaldehyde, and stained with red fluorescent wheat germ agglutinin (WGA) (Thermo Fisher Scientific) and DAPI (Sigma) to localize cell membranes and nuclei, respectively. All samples were then observed using a confocal microscope (SP5 Leica) with a 63× oil immersion objective.

### 2.13. Statistical Analysis

Quantitative results were reported as mean ± standard deviation (SD). Statistical comparisons were performed with a Student’s unpaired test. *p* values < 0.05 were considered statistically significant.

## 3. Results

### 3.1. Quantification, Intracellular Localization, and Cytotoxicity of Endocytosed and Shot Nanoparticles in hCMEC/D3 Cell Line

**Figure 1:**
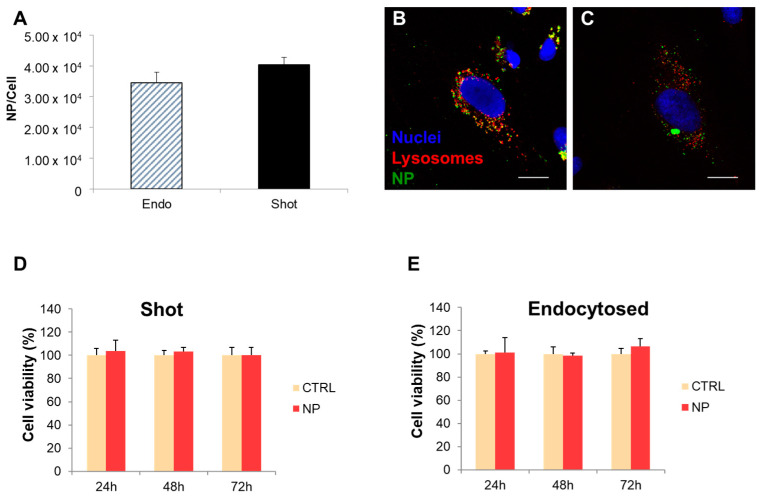
Quantification of endocytosed (endo) and shot 100 nm carboxyl-modified polystyrene nanoparticles (NPs) (**A**). About 3.6 × 10^10^ per cell were obtained for both endo and shot samples. Co-localization of NPs (green) with lysosomes (red) after endocytosis (**B**) and shooting (**C**). Blue are nuclei stained with DAPI. Scale bar 20 µm. Cytotoxicity assay results of shot (**D**) and endocytosed NPs (**E**). Results are expressed as mean percentage of cell viability normalized to non-treated control cells, considered as 100% of cell viability.

Quantification experiments were carried out for assessing the number of internalized NPs per cell, comparing the two different methods of internalization, namely endocytosis and gene gun. The ultimate goal was to obtain the same number of endocytosed and shot nanoparticles per cell in order to compare the effects of a different intracellular localization of NPs on cell response. Data showed that the concentrations to use for 100 nm NPs were 1.2 × 10^12^ NPs/mL for shot NPs and 3.6 × 10^10^ NPs/mL for endocytosed NPs in order to obtain the same number of NPs inside the cells (i.e., about 3.5 × 10^4^ NPs/cell) (Figure 1A). These concentrations were used for all the following experiments.

To verify that NPs were not cytotoxic for hCMEC/D3 cell line at all the experimental conditions used in this work, before other analyses, we performed Alamar blue assay. Cell viability was assessed up to 72 h on cells exposed to NPs through endocytosis and on cells shot with NPs and the results were compared to non-treated cells used as a control. Cell viability was determined by spectrofluorimetric analysis at 24, 48, and 72 h upon NP incubation. Experimental results showed that the cell viability was always around 100% for both endocytosed and shot nanoparticles until 72 h (Figure 1D,E), demonstrating that the NP endocytosis and shooting conditions did not affect cell viability.

Furthermore, we investigated the intracellular distribution of endocytosed and shot NPs in hCMEC/D3 by performing co-localization experiments with lysosomes. In particular, after 24 h from NP endocytosis and shooting in hCMEC/D3 cell line, an indirect immunofluorescence against the lysosomal marker LAMP2 was carried out. Endocytosed NPs clearly showed a perinuclear accumulation in the cytoplasm as large aggregates, and a significant co-localization with lysosomes after 24 h incubation (Figure 1B). Conversely, very small and few aggregates and no co-localization with lysosomes were observed for shot NPs (Figure 1C). Therefore, the use of the gene gun method allowed us to obtain free NPs in the cytoplasm in hCMEC/D3 cell line in agreement with previously reported results obtained in different cell lines [23].

### 3.2. Cellular Release of Endocytosed and Shot Nanoparticles

The accumulation across the endo-lysosomal pathway significantly reduces the ability of NPs to exit from the cells [19]. In fact, once in lysosomes, NPs are excreted with a low percentage. In this framework, we used the gene gun method to study the fate of NPs non-confined in vesicular structures and their excretion profile.

**Figure 2:**
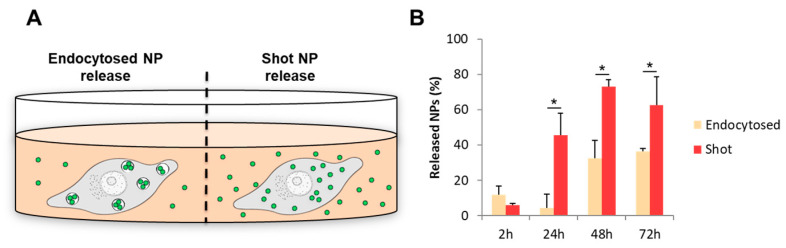
Schematic representation of the experimental conditions in standard culture of hCMEC/D3 cells (**A**). Quantification over time of released NPs from hCMEC/D3 cultured in standard conditions (**B**). * *p* < 0.05.

In order to point out the correlation between the intracellular localization and the fate of NPs, we investigated the profile of NP release from human brain endothelial cells (Figure 2A). Specifically, we first quantified the number of released NPs from hCMEC/D3 cells that were previously loaded with the same amount of endocytosed and shot NPs. The number of released NPs in cell culture media was measured at different timepoints, namely 2, 24, 48, and 72 h. Furthermore, the amount of NPs in the cell lysates at every timepoint was also determined. The number of released NPs was expressed as a percentage of the total number of NPs found in the cell lysate and cell culture medium. The results show that, for endocytosed NPs, the release already started after 2 h (Figure 2B). About 10% of NPs was found in the cell culture media. This phenomenon was probably due to the nanoparticles associated with the external part of the cell membrane and which were not internalized. In fact, after 24 h, only a slight and non-significant decrease in the release of endocytosed NPs was observed, supporting this hypothesis. Conversely, a significant increase in the percentage of released NPs was observed after 48 h. After 72 h, the percentage was kept constant likely due to an equilibrium between the number of NPs inside and outside the cells. For shot NPs, cell release was less than 10% after 2 h, similar to endocytosed NPs. However, contrary to endocytosed NPs, at 24 h, a significant increase in released NP percentage (>40%) was observed. The percentage of released NPs increased up to around 75% at 48 h and remained quite constant and higher than endocytosed NPs at 72 h (Figure 2B).

### 3.3. Cellular Release of Endocytosed and Shot Nanoparticles in the Basal Compartment of the BBB Endothelium

**Figure 3:**
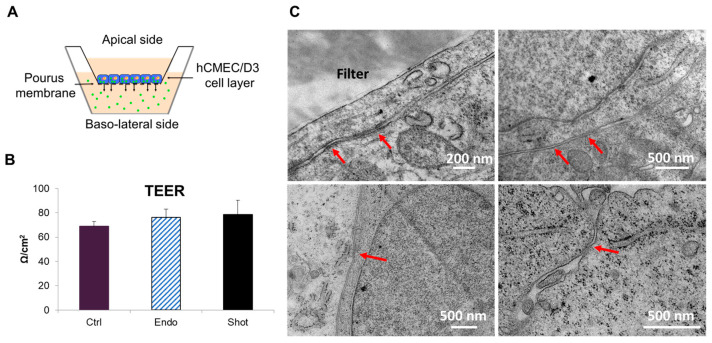
Schematic representation of Transwell system (**A**). TEER measurements after 72 h from cell seeding on Transwell filter indicating no significant difference among the different experimental conditions (**B**). Representative TEM images of hCMEC/D3 cells after 3 days of growth on Transwell supports according to the experimental conditions described in this work. Red arrows indicate tight junctions (**C**).

Starting from the above data, we performed the same experiment in order to study the effects of intracellular localization on NP excretion profile on a Transwell system mimicking the BBB endothelium (Figure 3A). In fact, brain endothelial cells are physiologically organized in a confluent layer that confers a polarization to the cells. In vitro, these cells can assume this phenotype when cultured on Transwell supports [34].

To address this issue, we seeded cells pre-incubated with endocytosed and shot NPs on a Transwell filter and we verified: (i) the effective formation of tight junctions, (ii) the intracellular localization of NPs, and (iii) the amount of released NPs by spectrofluorimetrc measurements.

The effective formation of tight junctions was assessed by transendothelial electrical resistance (TEER) measurements using a conventional epithelial volt-ohmmeter. The measured values of TEER, shown in Figure 3B, agreed with the values reported in the literature, at around 50 Ω·cm^2^ [35]. Here, we measured a value of around 65 Ω·cm^2^ in the presence of endocytosed and shot NPs. TEM analysis further confirmed the presence of tight junctions (Figure 3C), comparable to the same structures observed in brain capillary in vivo [36]. Therefore, we concluded that a confluent endothelial layer was correctly formed in our experimental conditions and that the presence of NPs did not alter the tight junction formation and integrity of the brain endothelium.

**Figure 4:**
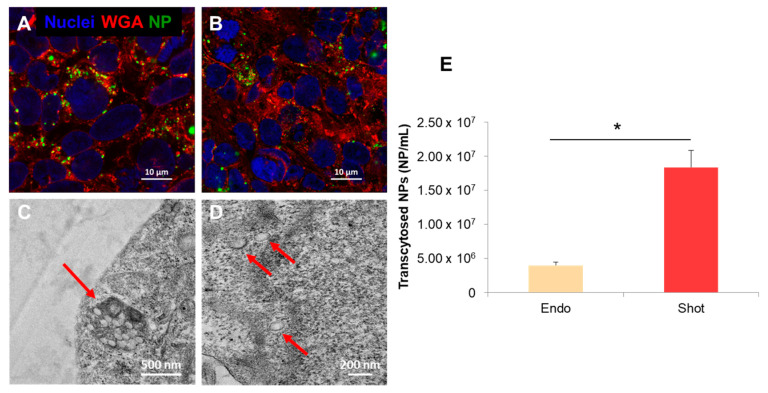
Confocal microscopy images of hCMEC/D3 cells on Transwell filter after 72 h from cell seeding on Transwell filter (**B**,**C**); endocytosed NPs (**A**) and shot NPs (**B**). Green: NPs; red: WGA-stained cell membranes; blue: DAPI-stained nuclei. TEM micrographs showing the intracellular localization of endocytosed (**C**) and shot (**D**) NPs. Endocytosed NPs are confined within endo-lysosomal compartment as large aggregates (**C**) (red arrow); shot NPs are single and free in the cytoplasm (**D**) (red arrows). Magnification bar 10 µm (**A**,**B**), 500 nm (**C**), and 200 nm (**D**). Quantitative analysis of nanoparticles transported across the BBB layer into the basolateral compartment (**E**). * *p* < 0.05.

The intracellular localization of endocytosed and shot NPs was also investigated by confocal microscopy and TEM analysis on Transwell filter. Figure 4A–D demonstrated the presence of NPs inside the cells. In particular, endocytosed NPs were confined in structures that were presumably multivesicular bodies (MVBs) (Figure 4C) and no free particles were visible in the cytosol (Figure 4D). Conversely, most of the shot NPs were freely dispersed in the cytoplasm and no aggregated NPs were observed. TEM observations were in agreement with confocal microscopy experiments of co-localization with lysosomes (Figure 3) and with a previous work where it was shown that shot NPs were present in cytoplasm [23].

Quantification experiments of transcytosed NPs were carried out in order to elucidate the effects of NP intracellular localization on the excretion profile of NPs. To address this issue, we performed spectrofluorimetric measurements after 72 h of culture in order to obtain the number of transcytosed NPs. The results agreed with the above experiments: in fact, the data indicated that shot NPs were able to reach the basal compartment more efficiently compared to endocytosed NPs. In particular, we found 4 × 10^6^ and 1.8 × 10^7^ NP/mL for endocytosed and shot NPs, respectively, in the basolateral (BL) compartment (Figure 4E). 

**Figure 5:**
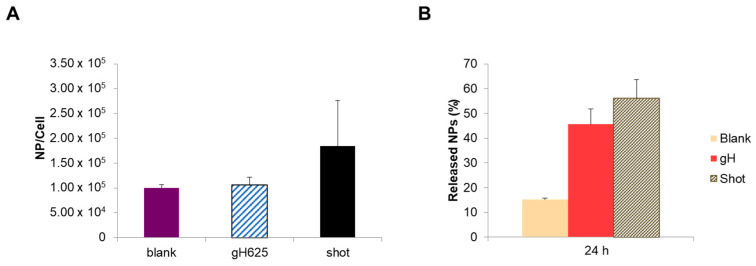
Quantification experiment of aminated and gH625-functionalized NPs (**A**). NP quantification experiments were carried out for assessing the number of internalized NPs per cell, comparing the blank NPs (blank), the gH625-functionalized NPs (gH625), and the shot aminated NPs (shot) (**A**). The ultimate goal was to obtain the same number of nanoparticles per cell in order to compare their effects on cell response. These concentrations were used in order to evaluate the excretion profile. Quantification of released NPs (**B**). We compared the excretion profile of blank, gH625, and shot NPs in order to compare the effects of different intracellular environments on NP cellular release. The data show that the percentage of gH and shot excreted NPs was similar. On the contrary, the percentage of endocytosed blank NPs was around 15%, which is significantly lower than gH625 NPs and shot NPs. This data further confirms that the accumulation of NPs inside the endo-lysosomes reduces the capability of NPs to leave the cells.

Furthermore, in order to confirm the role of different intracellular localization on NP excretion from hCMEC/D3 cells, we used aminated polystyrene NPs functionalized with the virus-derived peptide gH625 as a positive control (Figure 5). gH625 is a cell-penetrating peptide derived from a glycoprotein of the envelope of the Herpes virus Type 1 [29]. We previously reported that functionalization with the gH625 peptide affected the intracellular behavior of aminated polystyrene NPs, reduced endo-lysosomal accumulation, and improved BBB crossing in bEnd.3 cells [23]. As expected, the preliminary results reported in Figure 5B show that the gH625 functionalization allowed NPs to be released by the hCMEC/D3 cells more efficiently compared to the blank (non-functionalized) NPs, starting from the same initial NP intracellular content (Figure 5A). In fact, after 24 h, the percentage of released gH625-NPs was two times higher than blank NPs and comparable to shot NPs (Figure 5B). 

**Figure 6:**
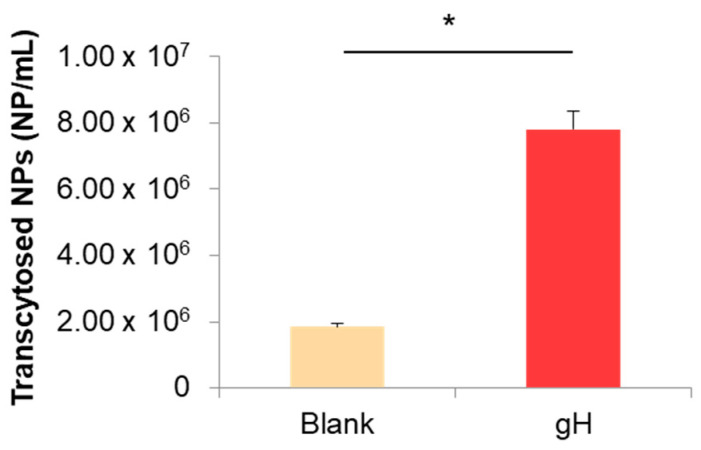
Quantification of transcytosed blank and gH625-NPs in Transwell system. * *p* < 0.05.

In addition, experiments carried out in Transwell systems indicated that the number of gH625-NPs transported to the basal compartment was four times higher than blank NPs (Figure 6). The gH625 functionalization determined the same effects in terms of hCMEDC/D3 cell excretion as shot NPs, thus confirming that reducing the endo-lysosomal accumulation of NPs improves their extracellular release. 

### 3.4. Characterization of Released Nanoparticles

In order to understand the effect of the different microenvironment experienced by NPs on their colloidal stability and protein corona formation, we performed dynamic light scattering (DLS) analysis and BCA assay. Results show that endocytosed NPs were excreted as large aggregates compared with shot NPs (Table 1). In fact, the hydrodynamic diameter measured for endocytosed NPs was four times higher compared to shot NPs. This result was due to the propensity of NPs to form aggregates during the fusion of endosomes for the formation of MVBs after the endocytosis process. Therefore, NPs were excreted after the fusion of endocytic vesicles with the cell membrane as aggregates. On the contrary, shot NPs were internalized from the cells as single entities and they rarely showed the propensity to form large aggregates in the cells (as shown in Figure 4C,D). Consequently, they could be excreted as single particles. 

**Figure 7:**
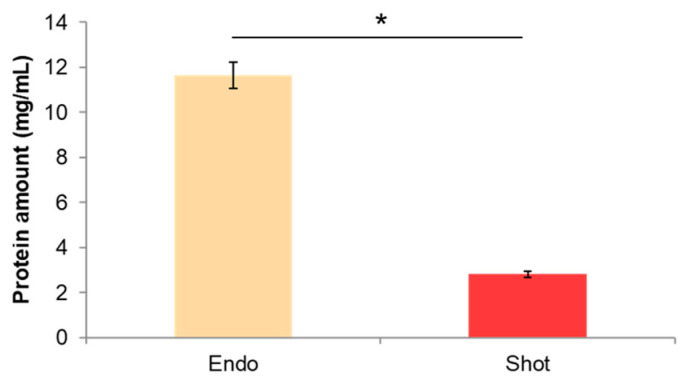
Amount of adsorbed proteins on endocytosed (endo) and shot NP surface after release from hCMEC/D3 cells determined by BCA assay. Data are reported as mean ± SD. * *p* < 0.05.

Furthermore, we measured the amount of proteins adsorbed on the surface of excreted NPs by BCA analysis and we found that the amount of proteins on the NP surface was higher for endocytosed NPs (Figure 7). This data suggested that the different intracellular experience affected the surface properties of NPs, which in turn, might change the NP–cell interactions with the target cells.

### 3.5. Internalization of Released Nanoparticles in Human Brain Tumor Cells

Once the BBB is crossed, NPs have to reach the brain parenchyma to exert their potential pharmacological action. Despite numerous studies reporting the presence of NPs in CNS in vivo [37,38], the effective uptake mechanisms of NPs in target cells remain elusive. 

**Figure 8:**
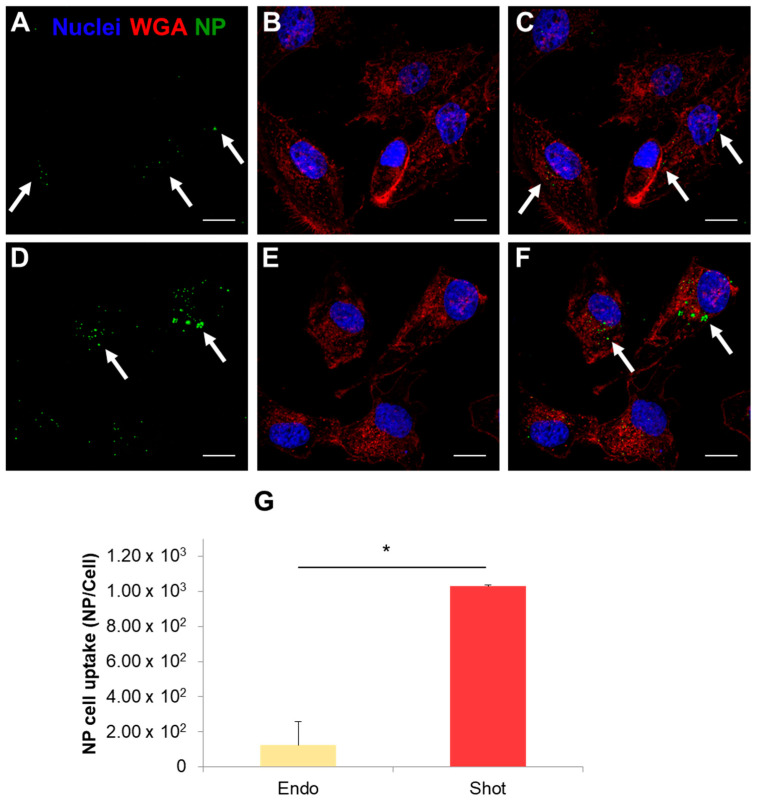
Uptake of NPs in U87 cell line after release from the BBB layer. Panels (**A**–**C**) indicate uptake of NPs endocytosed and then released by brain endothelial cells; panels (**D**–**F**) indicate uptake of NPs shot and then released by brain endothelial cells. Green: NPs; red: WGA-stained cell membranes; blue: DAPI-stained nuclei. (**A**,**D**) show green fluorescence channel; (**B**,**E**) show red and blue channels; (**C**,**F**) show the merge of the three fluorescence channels. White arrows indicate the internalized NPs. Magnification bar: 10 µm. Quantitative analysis of U87 cell uptake (**G**). Results indicate the number of internalized NPs per cell. * *p* < 0.05.

With the aim to study the fate of released NPs downstream of the BBB crossing, we treated brain tumor U87 cells with NPs released from the brain endothelial layer. In particular, U87 cells were incubated with the same concentrations of endocytosed and shot NPs released from the endothelial layer. Figure 8 shows the quantification of NPs internalized in U87 cells. Data demonstrated that NPs, released by BBB upon shooting, were taken up from target cells more efficiently compared to NPs previously endocytosed and released by BBB cells. More precisely, the amount of internalized NPs was 1.24 × 10^2^ NP/cell and 1.03 × 10^3^ NP/cell for endocytosed and shot NPs, respectively (Figure 8G). The quantitative analysis of the internalization of NPs pre-conditioned from the two different BBB-crossing mechanisms was further confirmed by confocal microscopy images (Figure 8A–F). In fact, the presence of cytoplasm-conditioned NPs (Figure 8D–F) was more evident compared to vesicle-conditioned NPs (Figure 8A–C). The former formed very small spots in the U87 cells while the latter formed larger and more evident aggregates within the cells.

## 4. Discussion

The fulfillment of the great potential of NPs for biomedical application, as nanosensors and nanocarriers, needs a complete knowledge of how they interact with cells, tissues, and organs. Despite numerous technological advancements and very interesting properties, very few nanomaterials have been approved by the Food and Drug Administration (FDA) for use in medical applications. Therefore, new efforts must focus on a detailed understanding of the interaction of nanomaterials with the cells at the fundamental level. There is proof that nanoparticles are indeed taken up by cells with an extent that depends on their physical–chemical properties and subsequent interactions. However, their subsequent release and/or intracellular localization/degradation, transfer to other cells, and/or translocation across tissue barriers are still unclarified and under investigation. In this work, we aimed at elucidating the behavior and fate of NPs as a function of their different intracellular localization, i.e., confined into endo-lysosomal compartment or free in the cytosol. We chose commercially available 100 nm carboxyl-modified polystyrene NPs as a model of fluorescently labelled nanoprobes. To obtain a different intracellular localization of NPs, we incubated human brain endothelial hCMEC/D3 cells with NPs dispersed in cell culture media or shot by gene gun method according to a procedure that we previously optimized and used for different cell types [23].

It is known that carboxyl-modified polystyrene NPs of different sizes did not exert cytotoxic effects on several cell types after internalization by endocytic mechanism [39,40]. Our results indicated that these NPs were not cytotoxic for human brain endothelial hCMEC/D3 cells in our experimental conditions, which is in agreement with the already reported observations on other cell types [39,40]. Moreover, the gene gun method did not affect cell viability as well, thereby demonstrating the feasibility of this method to deliver NPs directly inside the cytoplasm, and to allow in vitro fundamental studies of nano–bio interactions.

Previously, we demonstrated that, when exposed to the cell surfaces, amino-modified, 100 nm polystyrene NPs were taken up by mouse brain endothelial bEnd.3 cells through endocytic mechanisms and tended to accumulate in lysosomes [23,29]. Conversely, when NPs were allowed to bypass endocytic mechanisms, they were able to remain free in the cytoplasm and to not co-localize with lysosomes [23]. Similarly, in this work, we observed that when incubating carboxyl-modified 100 nm NPs with human brain endothelial hCMEC/D3 cells, most of them were internalized by endocytosis. Indirect immunofluorescence against the endo-lysosomal marker LAMP2 indicated co-localization of endocytosed NPs with these intracellular compartments. Conversely, shot NPs were free in the cytoplasm, as evidenced by TEM images.

The endocytosis of nanomaterials has been widely investigated in recent years. However, only a limited number of reports show the exocytosis of NPs. In general, exocytosis processes occur via the fusion of vesicular membranes with the plasma membrane, leading to the release of the vesicular content into the extracellular environment [41]. Exocytosis can follow two pathways in the cell: constitutive and regulated [42,43]. Constitutive exocytosis is present in all cell types; the regulated pathway is observed in cells that are specialized in secreting their products on demand. It has been reported that endocytosis and exocytosis of nanomaterials are coupled and can influence each other by stimulating or compensating [41]. Moreover, exocytosis occurs more slowly than endocytosis and it is affected by several parameters such as cell type, NP properties (i.e., size, surface charge, surface functionalization, etc.), NP dose, and exposure time [24]. 

Our results indicated that endocytosed NPs were excreted by human brain endothelial cells. Their release was slower and fewer than positive NPs used in previously reported works [41]. These observations confirmed that the excretion of nanomaterials depends on NP type. Furthermore, our data indicated that cytoplasmic nanoparticles (shot NPs) were expelled faster than the same endocytosed NPs. In particular, the percentage of endocytosed NPs that were able to leave the cells was lower than shot NPs at 24, 48, and 72 h, demonstrating that internalized NPs that bypassed the endo-lysosomal compartment were excreted more efficiently. After 48 h, the release percentages of both endocytosed and shot NPs reached a plateau, probably due to a balance between NP excretion and the cellular re-absorption of released NPs. Taken altogether, these results showed that the intracellular localization of NPs affects their retention. The hypothesis is that NPs enclosed in endo-lysosomal vesicles are less able or slower to leave the cells compared to cytosolic NPs because the exocytic vesicles (with cargo) must be formed/separated from multivesicular bodies, which are formed following the fusion of the endosomes, and transported to the cell membrane to be excreted. In line with our observations and in spite of the current sparse knowledge on the mechanisms of NP exocytosis, Liu et al. reported that the lysosomal pathway is not the main exocytosis mechanism used by NPs to be excreted from several cell types despite the prevalent NP accumulation into the lysosomes [30].

Transwell experiments were carried out with the aim to work in a more physiologically relevant in vitro model of BBB and to achieve information about BBB crossing by NPs. Results obtained in Transwell systems demonstrated that the intracellular localization of NPs also influenced NP transport and release across the BBB layer. The endocytosis process of carboxylated NPs unavoidably leads to the accumulation into lysosomal compartment as widely described in literature [39]. Lysosomes are a terminal degradative compartment of the endocytic pathway, hence, transport into lysosomes is considered mainly unidirectional [44,45]. Even if lysosome fusion with the plasma membrane is a process present in several specialized cell types [46], the accumulation in lysosomes reduces the ability of NPs to reach the basolateral side of the BBB in vitro [19,47], thus diminishing the transport of NPs to the CNS. On the contrary, by bypassing the lysosomal compartment, we demonstrated that NPs are able to reach the basal compartment more efficiently. Therefore, using strategies that help NPs to escape the endo-lysosomal compartment ameliorates NP transcytosis. In this context, we previously reported that the functionalization with the virus-derived cell-penetrating peptide gH625 affected the intracellular behavior of aminated polystyrene NPs, reduced endo-lysosomal accumulation, and improved BBB crossing in bEnd.3 cells [23]. Furthermore, by avoiding lysosomal storage, the presence of gH625 peptide reduced the cytotoxic effects of 50 nm aminated polystyrene NPs in several cell types [33]. Based on these results, in this work, we used gH625-functionalized NPs as a positive control to confirm the role of different intracellular localization on NP excretion from hCMEC/D3 cells (Figure 5 and Figure 6). Our preliminary results showed that the gH625 functionalization allowed NPs to be released by the hCMEC/D3 cells more efficiently compared to the blank (non-functionalized) NPs, starting from the same initial NP intracellular content. In fact, after 24 h, the percentage of released gH625-NPs was two times higher than blank NPs and comparable to shot NPs. In addition, experiments carried out in Transwell systems indicated that the number of gH625-NPs transported to the basal compartment was four times higher than blank NPs. The functionalization with gH625 peptide determined the same effects in terms of hCMEDC/D3 cell excretion as shot NPs, thus confirming that reducing the endo-lysosomal accumulation of NPs improves their extracellular release. 

Uptake and lysosomal processing can modify NP surface, thus likely changing the surface properties of the exocytosed NPs. As a consequence of such modification, exocytosed NPs may recognize different targets or receptors on the cell membrane influencing their long-term fate [24,31]. In this framework, we analyzed the uptake of endocytosed and shot NPs after their release from hCMEC/D3 cells in human brain U87 cells. Our data indicated that endocytosed NPs were internalized to a lesser extent in U87 cells than shot NPs. This result was probably due to the propensity of endocytosed NPs to leave the cells as large aggregates (as demonstrated from DLS analysis of excreted NPs). The aggregation of NPs because of the uptake mechanism unavoidably led to a physical hindrance and, hence, a greater difficulty to be internalized by target cells. Moreover, as shown by BCA assay, released NPs showed a different amount of adsorbed proteins depending on the different internalization mechanism (Figure 7). In particular, endocytosed NPs adsorbed a greater amount of proteins upon hCMEC/D3 release than shot NPs. These observations suggest that the different intracellular compartments experienced by endocytosed and shot NPs also affected NP surface in terms of protein corona formation and, likely, composition. These findings are in agreement with Xiao et al., showing that BBB crossing changed the composition of the protein corona of transferrin (Tf)-functionalized NPs as well as their targeting capabilities [32]. Because the NPs used in this work were not functionalized with targeting moieties and analyzing confocal images of Figure 7, we hypothesized that exocytosed NPs were likely internalized by U87 cells through endocytosis and, hence, accumulated into the endo-lysosomal compartment. This aspect should be considered to design effective NPs for relevant clinical applications by creating multistage NPs (e.g., with a pH-sensitive shell functionalized with molecules that allow BBB targeting and crossing—by avoiding endo-lysosomal confinement—and a drug-loaded core functionalized with specific targeting moieties).

The results of this work open interesting new questions about the nano–bio interactions to identify the underlying mechanisms that regulate NP exocytosis and about the nanomaterial design to boost their theragnostic potential. In this direction, further investigations are necessary to elucidate biological mechanisms involved in the excretion of NPs not confined in the endo-lysosomal compartments.

## 5. Conclusions

In conclusion, our results indicated that the intracellular localization of NPs affects their extracellular release kinetics as well as their aggregation state upon excretion. Consequently, the uptake of excreted NPs in tumor target cells is influenced. This work highlights the importance of studying the mechanisms of nano–bio interactions and, in particular, the long-term fate of NPs to predict their behavior and to boost their theragnostic potential. NPs present some limits due to the accumulation along endo-lysosomal pathway, which decreases the efficiency of BBB crossing, thus also leading, in some cases, to cytotoxic effects. We demonstrated that bypassing the endo-lysosomal compartment improves NP extracellular release and tumor cell uptake. This aspect is particularly relevant to design and to fully explore the potential use of NPs as nanosensors and/or nanocarriers for the diagnosis and treatment of brain diseases.

Our findings open new questions about the biological mechanisms underlying the excretion of NPs as well as the protein corona evolution around NPs that experience different intracellular environments.

## Figures and Tables

**Table 1:** Size, polydispersity index (PDI), and Z-potential of NPs in different experimental conditions and dispersion media.

	Size (nm ± SD)	PDI	Ζ-Potential (mV ± SD)
ddH_2_0	108 ± 1.1	0.005 ± 0.003	−48.3 ± 0.9
EBM-2 w/o FBS	181.7 ± 2.5	0.271 ± 0.015	−38.7 ± 0.8
EBM-2 + 10% FBS	210.2 ± 2.5	0.252 ± 0.016	−30.3 ± 0.7
EBM-2 endo	879.5 ± 147.9	0.723 ± 0.113	−32.6 ± 1.5
EBM-2 shot	205.7 ± 11.2	0.113 ± 0.088	−37.2 ± 0.5

## Data Availability

Data are available from the authors upon reasonable request.
